# Population cigarette consumption in Great Britain: novel insights using retail sales data

**DOI:** 10.1186/s12889-017-4950-z

**Published:** 2017-12-20

**Authors:** Mark Robinson, Garth Reid

**Affiliations:** Public Health Science Directorate, NHS Health Scotland, Glasgow, Scotland

## Abstract

**Background:**

Accurate data to measure population cigarette consumption are vital for surveillance and for evaluating the impact of tobacco control policies. This study uses cigarette retail sales data to provide novel insights into trends and patterns in cigarette consumption in Great Britain.

**Methods:**

Cigarette sales estimates derived from electronic sales from most large grocery stores and a weighted representative sample of smaller convenience stores were obtained from Nielsen. Data on the number of cigarette sticks sold per calendar month and per week were obtained for Scotland and England/Wales (combined) for the period January 2008 to December 2015. Cigarette consumption per adult smoker per month was calculated using survey-based smoking prevalence estimates and mid-year population estimates.

**Results:**

Population cigarette consumption in Great Britain declined between 2008 and 2013. Cigarette sales have since stabilised at around 400 cigarettes per adult smoker per month. Cigarettes sold in 14- to 19-packs have substituted a sharp decline in 20-packs and now account for over half of all cigarettes sold in Great Britain. Cigarette consumption has been consistently higher in Scotland than England/Wales. This is due to higher sales of 20-packs in Scotland between 2008 and 2013, which has been substituted by higher sales of 14- to 19-packs in recent years.

**Conclusions:**

Cigarette retail sales data provide unique insights into levels and patterns of cigarette consumption and should be used for monitoring and evaluating tobacco control policy in Great Britain.

**Electronic supplementary material:**

The online version of this article (10.1186/s12889-017-4950-z) contains supplementary material, which is available to authorized users.

## Background

In Great Britain, one in five adults smoke [1] with smoking accounting for almost 100,000 deaths per year [2]. Accurate data with which to measure patterns and levels of cigarette consumption in a population are vital to evaluate the impact of such tobacco control policies and to monitor trends over time [3, 4]. In Great Britain, this is done almost exclusively using self-reported survey data [5, 6], despite well-known limitations related to sampling design, low response rates and recall bias [7]. Sales data are generally considered to be a more robust alternative for estimating population levels of health behaviours, including smoking, because they are objective [3, 7, 8]. While cigarette retail sales data have been used in a number of countries and settings [9, 10], their use in Great Britain remains very limited. Taxation data can be used to estimate average population cigarette consumption, but these UK-level data cannot be disaggregated to constituent countries and publicly available data contain limited information on pack size, price and place of sale.

In a recent study, we assessed the validity and reliability of retail sales data for estimating cigarette consumption in Scotland [11]. It was concluded that cigarette retail sales data offer the potential to strengthen national monitoring and policy evaluation when used alongside data for other smoking-related indicators and on the proviso that biases potentially affecting their robustness are carefully considered. The purpose of this brief paper is to use retail sales data to present, for the first time, trends and patterns in population cigarette consumption in Great Britain.

## Methods

### Cigarette sales data

Cigarette retail sales data were obtained from the market research company, Nielsen, who approved the use and publication of their data by NHS Health Scotland for the purpose of measuring population cigarette consumption. Nielsen uses electronic point of sale (EPoS) data to estimate retail sales. For large grocery retailers, including most of the large supermarket chains, census retail sales data are collected by Nielsen on a weekly basis. The data comprise scanned readings at EPoS of the type and volume of each pack of cigarettes sold, and a net retail price. The Nielsen sampling frame excludes discount retailers Aldi and Lidl, though these retailers do not sell cigarettes. For remaining outlets, which are usually smaller and used for impulse and top-up purchases (hereafter ‘convenience stores’), retail sales are estimated using EPoS data provided by a weighted stratified random sample. Manual audits are required to be undertaken by Nielsen in some independent outlets (a sub-group of convenience stores), where auditors visit retailers to examine invoices and other key sources of data. The aim of this approach is to enhance representativeness from stores that do not collect EPoS data. The sample is designed to be representative for Great Britain as a whole and separately for Scotland and England/Wales (combined). It has been estimated that Nielsen capture 87% of total grocery market sales in Great Britain [12].

Data on the number of cigarette sticks sold per calendar month by cigarette pack size were obtained separately for Scotland and England/Wales for the period January 2008 to December 2015. Pack sizes included 10-, 14-, 17-, 18-, 19- and 20-packs; 14- to 19-packs were collapsed into a single category in the results for clarity of presentation though changes by individual category are described where appropriate. Data for Great Britain overall were also available by retailer category. Weekly cigarette sales data by pack size were also obtained for the period April 2011 to December 2015; only sales by large grocery retailers were available at weekly level.

### Data analysis

To account for between-country differences in smoking prevalence (see Additional file 1) and population size, we expressed the cigarette sales data as ‘cigarette consumption per adult (aged 16+ years) smoker’. Adults were defined as those aged 16+ years for consistency with smoking prevalence estimates obtained from national population health surveys even though the legal age of smoking in Great Britain is 18 years. We obtained annual estimates of self-reported smoking prevalence in 2007–2014 for Scotland, England/Wales (combined) and Great Britain overall from the Office for National Statistic’s Opinions and Lifestyle Survey [1, 13]. Annual mid-year population estimates were obtained from the ONS [13] and National Records of Scotland [14]. Weekly and monthly smoking prevalence and population estimates were calculated for the time period January 2007 to December 2015 by fitting linear and polynomial curves to the annual prevalence estimates (which were assumed to be mid-year) in Microsoft Excel 2013. The best-fitting curve for each was selected based on the coefficient of determination (R^2^).

## Results

### Trends in population cigarette consumption, Scotland and England/Wales

**Fig. 1:**
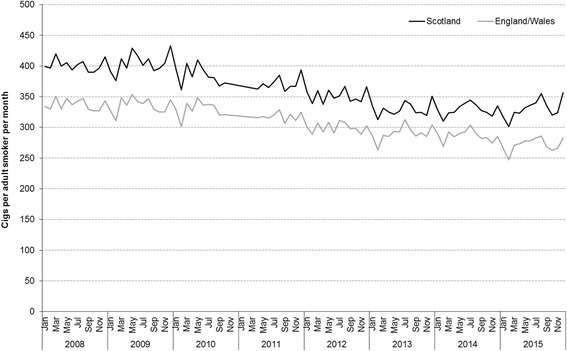
Trends in mean monthly cigarette sales per adult smoker, Scotland and England/Wales, 2008–2015. Footnote: Cigarette sales data were obtained from Nielsen

Population cigarette consumption in Scotland was broadly stable between 2008 and early 2010, fluctuating around 400 cigarettes per adult smoker (Fig. 1). This was followed by a fluctuating downward trend to early 2013. Cigarette consumption then remained broadly stable to December 2015 at around 330 cigarettes per adult smoker. In October–December 2015, 17% fewer cigarettes were sold per adult smoker in Scotland than in the same time period in 2008. Levels of population cigarette consumption were consistently higher in Scotland than in England/Wales over the study time period; however, trends over time have been similar across Great Britain. Although the downward trend since 2010 was faster in Scotland meaning levels converged by 2013, the downward trend in England/Wales continued to December 2015 (albeit at a slower rate) in contrast to the plateaux in Scotland. As a result, the difference between Scotland and the rest of Great Britain in October–December 2015 was similar to that observed in the same time period in 2008.

### Trends in population cigarette consumption, by pack size

**Fig. 2:**
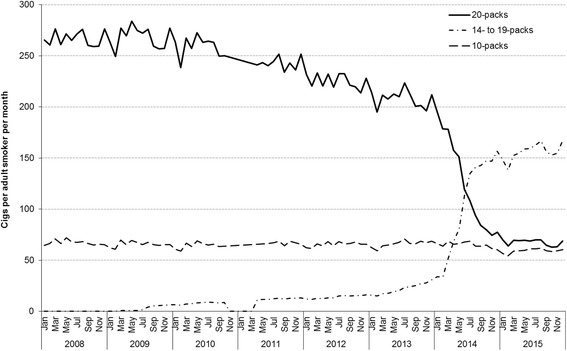
Trends in mean monthly cigarette sales per adult smoker, by pack size, Great Britain, 2008–2015. Footnote: Cigarette sales data were obtained from Nielsen

Between 2008 and 2013, cigarettes sold in 10- and, in particular, 20-packs dominated the cigarette retail market in Great Britain (Fig. 2). However, the overall decline in total cigarette sales has been almost entirely driven by sales of 20 packs, which fell by 76% in Great Britain between Oct-Dec 2008 and Oct-Dec 2015. There was also a 9% decline in the number of cigarettes sold per adult per month in 10 packs over the same time period. The downward trend in 10- and 20-packs has been partly offset by a rising trend in cigarettes sold in 14- to 19-packs.

Sales of 19-packs emerged in April 2009 and increased gradually to March 2013 (see Additional file 2). The upward trend in sales of 19-packs steepened from April 2013 before increasing rapidly between March and December 2014. There has since been a decline, partly offset by growth in 18-packs, but by the end of the study time period 19-packs still held the largest market share (38% in Scotland; 28% in England/Wales). Compared with England/Wales, 20-packs accounted for the higher total sales in Scotland between 2008 and 2013, but this pattern has been substituted by higher sales of 14- to 19-packs in 2014 and 2015 (Additional file 2).

### Trends in population cigarette consumption, by retailer type

**Fig. 3:**
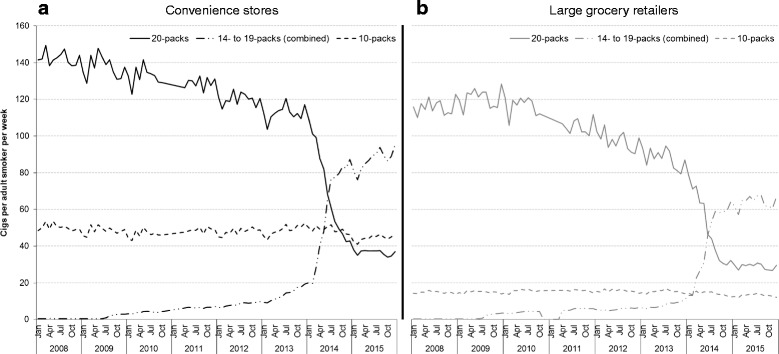
Trends in mean monthly cigarette sales per adult smoker in (a) large grocery retailers and (b) convenience stores, by pack size, Great Britain, 2008–2015. Footnote: Cigarette sales data were obtained from Nielsen

Cigarettes sold through convenience stores accounted for between 55 and 60% of total cigarette sales in Great Britain between November 2007 and December 2015 (Fig. 3). A higher number of cigarettes were sold per adult in 20-packs through convenience stores over the entire study time period, with a downward trend in sales being observed across both retailer categories (although the downward trend in sales through large grocery retailers began after the downward trend in convenience stores). The most notable difference between retailer categories is sales of 10-packs. Over the time period analysed, 10-pack sales in convenience stores have been consistently 3–4 times higher than in large grocery retailers. Recent trends also suggest that the growth in 14- to 19-packs has been higher in convenience stores.

### Trends in weekly population cigarette consumption

**Fig. 4:**
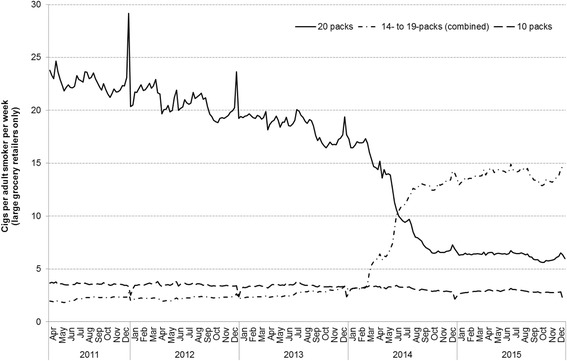
Trends in mean weekly cigarette sales per adult smoker, by pack size, Great Britain, 2011–2015. Footnote: Cigarette sales data were obtained from Nielsen

Weekly cigarette sales data were available from April 2011 and provide a more granular insight into population cigarette consumption, particularly seasonal patterns. The data reveal a sharp increase in cigarette sales in Great Britain in late December of each year, though the extent of the increase has diminished over time (Fig. 4). The rise over the Christmas period is driven by higher sales of cigarettes in 20-packs, with a corresponding decline in sales of 10-packs. Broadly similar patterns were observed when weekly cigarette sales data were analysed by country (see Additional file 3).

## Discussion

This paper has presented trends in population cigarette consumption in Great Britain using objective retail sales data. The analysis has shown that population cigarette consumption declined between 2008 and 2013 before stabilising. Cigarettes sold in 14- to 19-packs have substituted a sharp decline in 20-packs and now account for over half of all cigarettes sold in Great Britain. Cigarette consumption has been consistently higher in Scotland than England/Wales. This was due to higher sales of 20-packs in Scotland between 2008 and 2013, but has been substituted by higher sales of 14- to 19-packs in recent years. Convenience stores account for the slight majority of total cigarette sales in Great Britain with notably higher sales of 10-packs than large grocery retailers.

A key factor contributing the downward trend in population cigarette consumption in Great Britain as indicated by retail sales data is likely to be rising cigarette prices [15, 16]. Indeed, price is also likely to explain the rise of cigarettes sold in 19-packs. Manufacturers have made smaller pack sizes available to enable brands to maintain specific price points [17]. In addition, lower priced or ‘value’ brands have become increasingly popular among consumers [18]. This may, indirectly, have contributed to a greater reduction in cigarette sales than would have been expected based on the downward trend in sales of 20-packs before 19-packs emerged into the market. If it assumed that sales of 19-packs have directly replaced sales of 20-packs, 350 million fewer cigarettes were sold in Great Britain over the 5-year period March 2009–March 2014 due to a reduction in pack size. This is equivalent to 17% of the fall in total cigarette sales. More advanced statistical modelling of the data may help to strengthen this interpretation. Indeed, the availability of the cigarette retail sales data may be useful in evaluating the impact, if any, of the introduction of standardised packaging as well as the new EU regulations to introduce a minimum pack size of 20 cigarettes.

The rapid growth in the popularity of electronic cigarettes (‘e-cigarettes’) in Great Britain may also have contributed to the overall decline in population cigarette consumption since 2008. This assertion is supported by the results of Beard et al. [19] who showed that the prevalence of e-cigarette use by smokers in England increased from 2% in 2011 to 21% in mid-2013 with a stable trend thereafter. If it is assumed that smokers who start using e-cigarettes reduce the number of cigarettes they smoke [20, 21] this trend is consistent with those presented in Fig. 1: mean number of cigarettes sold per adult smoker declined between 2011 and 2013, with stability thereafter. Robust data on e-cigarette sales would help to substantiate this assertion but are currently lacking.

The specific reasons behind the consistently higher level of cigarette consumption in Scotland compared with England/Wales are unclear. The retail environment across Great Britain is similar with the same major retailers (supermarkets and chains of smaller stores) and tobacco multinationals operating in both jurisdictions. The policy context is also similar. For example, Scotland banned smoking in public places in 2006, with England/Wales introducing the same legislation in 2007. In addition, the increase in the minimum age of purchase to 18 years was brought into force within a day of each other (30th September 2007 in Scotland and 1st October in England/ Wales). However, besides the retail and policy environments, cigarette consumption patterns are influenced by a wide range of other factors, including historical smoking rates, demographics, cultural norms and socioeconomic deprivation. Given the strong social patterning of smoking [5], comparing Scotland with subnational areas of England/Wales with a more similar social and deprivation profile may have narrowed the differences observed [22, 23].

### Strengths and limitations

This is the first time that retail sales data have been used to assess trends in population cigarette consumption and to explore differences between constituent countries in Great Britain. Using routinely available data on population size and smoking prevalence, we were able to express population consumption as cigarettes per adult smoker, thereby strengthening our interpretation. The cigarette sales data were available at frequent time periods (monthly and for more recent data weekly) with breakdowns by pack size. This enabled novel insights into market share changes. Data at Great Britain level were also available by retailer category and these revealed differences in the number of cigarettes sold in different pack sizes between large grocery retailers and smaller, convenience stores.

These features of cigarette retail sales data show the usefulness of the data for policy monitoring and evaluation. Indeed, data on cigarettes either cleared for sale or sold by retailers have been used to evaluate tobacco control efforts in other countries (for review see [11]). In Great Britain, recent tobacco control polices have included a ban on smoking in public places and a tobacco display ban for retailers selling tobacco products. Legislation to introduce standardised (or ‘plain’) packaging was introduced in May 2016 with tobacco manufacturers given one year to achieve full compliance [24]. The legislation coincided with the introduction of the European Union (EU) Tobacco Products Directive which imposes new regulations on how tobacco products are manufactured, produced and presented in the EU, including minimum content for unit packs and increased size of health warnings [25]. Cigarette sales data offer the potential to strengthen the evaluation of these recent legislative and regulatory controls in Great Britain.

The most important limitation of using retail sales data is their vulnerability to biases that can impact on their validity and reliability. This includes, for example, measurement error associated with Nielsen’s data collection methods, such as the non-inclusion of outlets that sell cigarettes and non-response bias from outlets invited to be part of their sample [11]. The size of this bias is not quantifiable; however, levels and trends of annual population cigarette consumption derived from Nielsen data are broadly consistent with those based on tax clearance data, which provides reassurance that the data are representative and measure what they purport to measure [11]. Furthermore, Nielsen data are now generally considered the gold standard source of data for monitoring and evaluating population alcohol consumption in Great Britain [7, 26]. This followed a detailed critique of the sampling methods used by Nielsen to estimate alcohol sales [27], which are very similar for cigarette sales [11]. Although some methods may be considered commercially sensitive, developing good working relationships with data providers will help others to enhance the understanding and interpretation of cigarette sales data in their own context.

Other important biases affecting the validity and reliability of cigarette sales data were identified in a recent report [11]. Using the best available data, it was estimated that cigarette retail sales data are likely to have underestimated actual population cigarette consumption in Scotland in 2013 by 15%, with a wide range of uncertainty around this estimate and notable changes over time. This was largely due to the consumption of illicit cigarettes, cross-border purchases and duty-free shopping. These biases present a challenge to the use of sales data to estimate population cigarette consumption. Nonetheless, we believe that the novel insights presented in this paper provide support for our earlier assertion that sales data offer the potential to strengthen the monitoring and evaluation of tobacco control policy when triangulated with other sources of data [28].

A final limitation is that the sales data presented here include cigarette sales only. As such, it is not possible to comment in trends in overall tobacco consumption. For example, there has been a recent growth in the consumption of loose, or ‘roll-your-own’, tobacco and this will have offset some of the overall decline in the consumption of manufactured cigarettes [29].

## Conclusion

This study is the first to present trends and patterns in population cigarette consumption in Great Britain using retail sales data. The granularity of the sales data has enabled novel trends and patterns to be identified and we suggest they will be useful for future monitoring and evaluation of tobacco control policy in Great Britain and beyond.

## Additional files

Additional file 1: Cigarette smoking prevalence in Scotland and England/Wales, 2007–2014 (DOCX 16 kb)

Additional file 2: Trends in mean monthly cigarette sales per adult smoker, by pack size, Scotland and England/Wales, 2008–2015 (DOCX 71 kb)

Additional file 3: Trends in mean weekly cigarette sales per adult smoker, by pack size, Scotland and England/Wales, 2011–2015 (DOCX 77 kb)
