# Supplementary material for: Population cigarette consumption in Great Britain: novel insights using retail sales data
Source: BMC Public Health. 2017 Dec 20;17:941. doi: 10.1186/s12889-017-4950-z (PMC5738187; doi:10.1186/s12889-017-4950-z)
Supplement: Supplementary file 1 — Cigarette smoking prevalence in Scotland and England/Wales, 2007–2014 (DOCX 16 kb) [file 12889_2017_4950_MOESM1_ESM.docx]

**Additional File 1**

**Cigarette smoking prevalence in Scotland and England/Wales, 2007-2014**

|  | **Smoking prevalence (%)** | |
| --- | --- | --- |
|  | **Scotland** | **England/Wales** |
| **2007** | 23.9 | 20.6 |
| **2008** | 23.7 | 20.8 |
| **2009** | 24.5 | 20.7 |
| **2010** | 24.5 | 19.9 |
| **2011** | 24.6 | 19.4 |
| **2012** | 24.2 | 20.1 |
| **2013** | 23.0 | 18.8 |
| **2014** | 21.4 | 18.5 |

Source: ONS Opinions and Lifestyle Survey
