# Supplementary material for: Population cigarette consumption in Great Britain: novel insights using retail sales data
Source: BMC Public Health. 2017 Dec 20;17:941. doi: 10.1186/s12889-017-4950-z (PMC5738187; doi:10.1186/s12889-017-4950-z)
Supplement: Supplementary file 2 — Trends in mean monthly cigarette sales per adult smoker, by pack size, Scotland and England/Wales, 2008–2015 (DOCX 71 kb) [file 12889_2017_4950_MOESM2_ESM.docx]

**Additional File 2**

**Trends in mean monthly cigarette sales per adult smoker, by pack size, Scotland and England/Wales, 2008-2015**


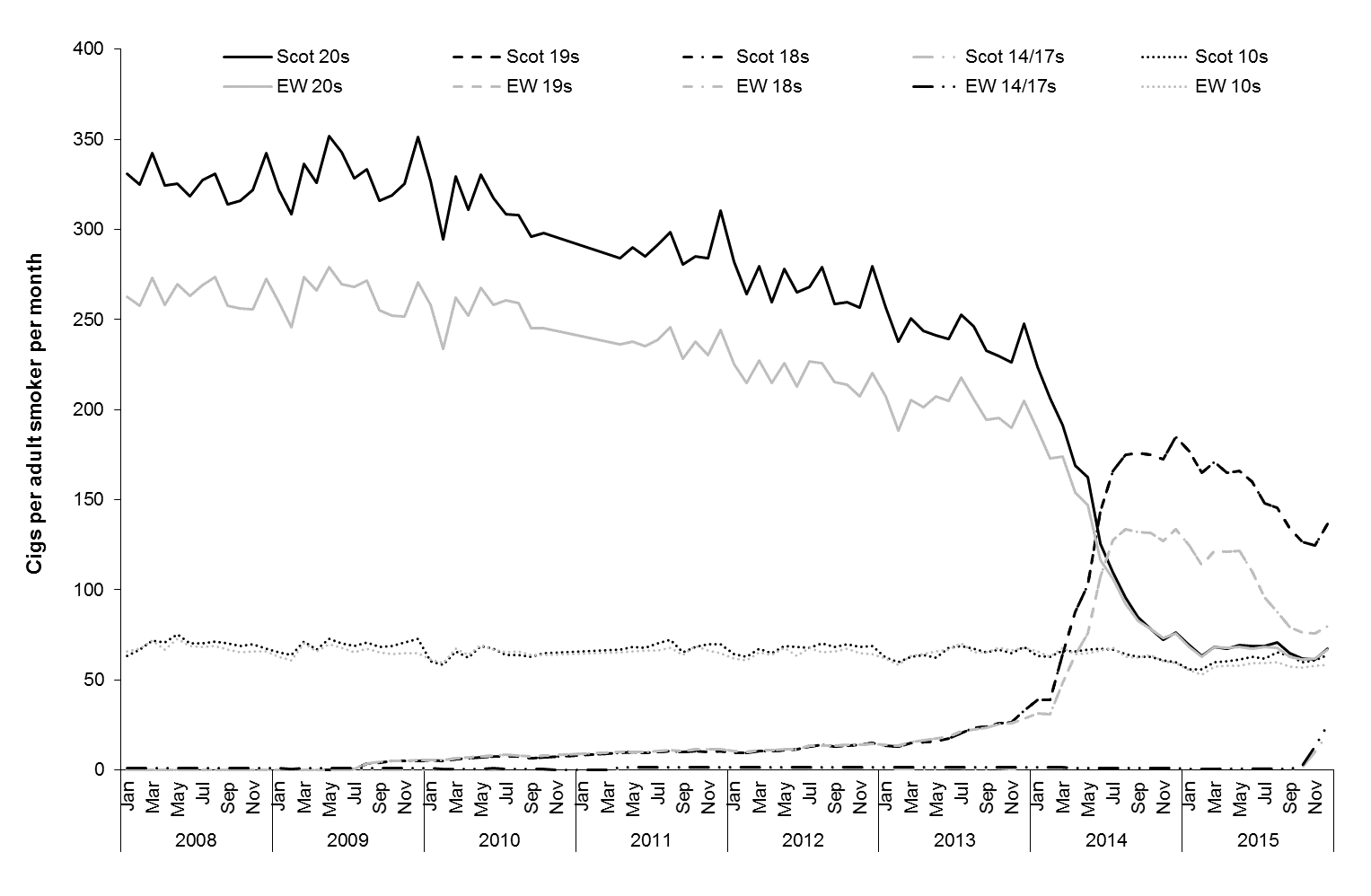


Source: Cigarette sales data were obtained from Nielsen. Notes: Scot = Scotland; EW = England/Wales
