# Supplementary material for: Population cigarette consumption in Great Britain: novel insights using retail sales data
Source: BMC Public Health. 2017 Dec 20;17:941. doi: 10.1186/s12889-017-4950-z (PMC5738187; doi:10.1186/s12889-017-4950-z)
Supplement: Supplementary file 3 — Trends in mean weekly cigarette sales per adult smoker, by pack size, Scotland and England/Wales, 2011–2015 (DOCX 77 kb) [file 12889_2017_4950_MOESM3_ESM.docx]

**Additional File 3**

**Trends in mean weekly cigarette sales per adult smoker, by pack size, Scotland and England/Wales, 2011-2015**


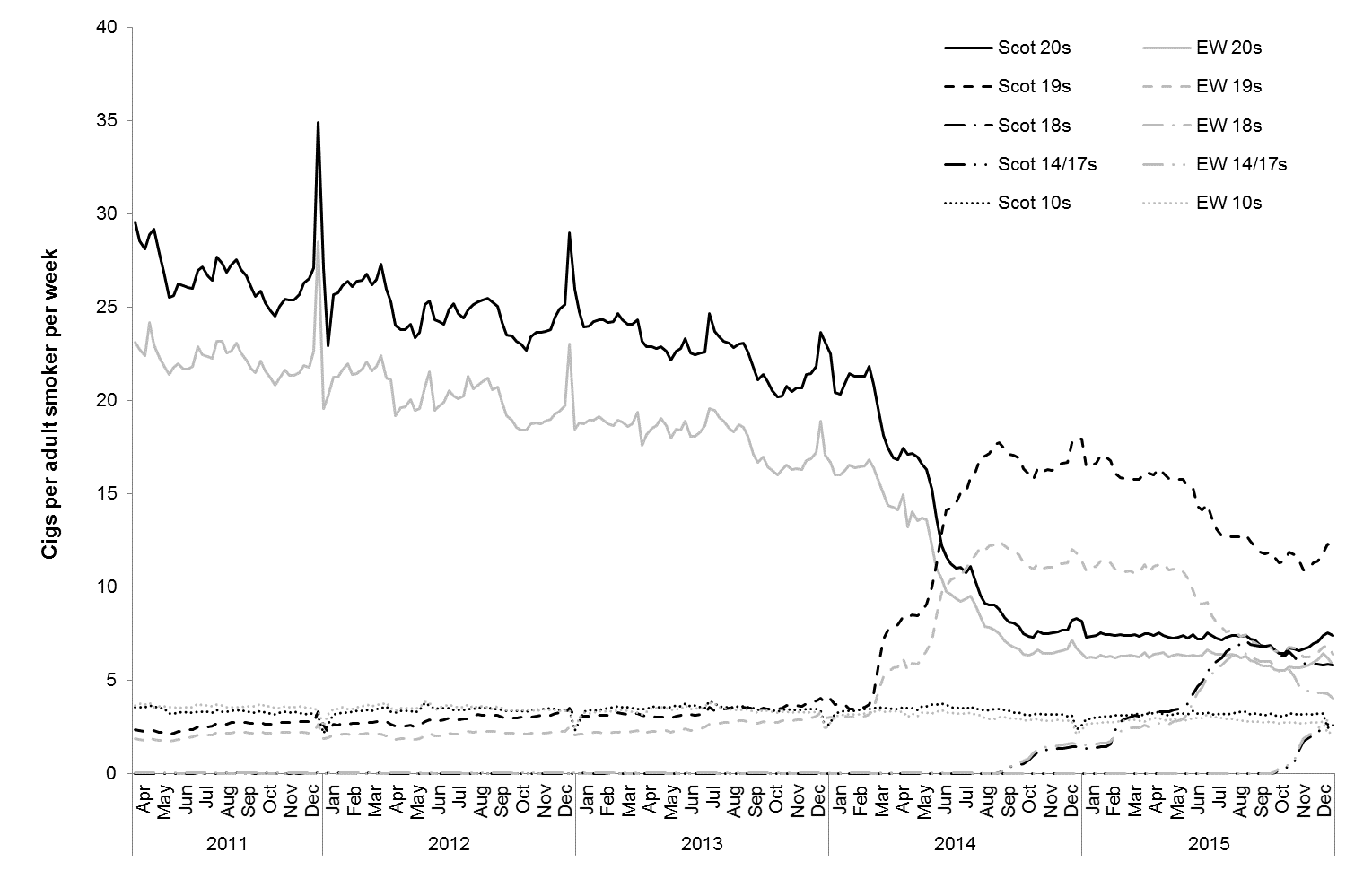


Source: Cigarette sales data were obtained from Nielsen. Notes: Scot = Scotland; EW = England/Wales
